# Phosphorus mobilizing consortium Mammoth P^™^ enhances plant growth

**DOI:** 10.7717/peerj.2121

**Published:** 2016-06-14

**Authors:** Peter Baas, Colin Bell, Lauren M. Mancini, Melanie N. Lee, Richard T. Conant, Matthew D. Wallenstein

**Affiliations:** 1 Growcentia, Inc., Fort Collins, CO, United States; 2 Natural Resource Ecology Laboratory, Colorado State University, Fort Collins, CO, United States

**Keywords:** Phosphorus mobilization, Bacteria, Tomato, Jalapeño, Herbs, Turf grass, Wheat

## Abstract

Phosphorus (P) is a critical nutrient used to maximize plant growth and yield. Current agriculture management practices commonly experience low plant P use efficiency due to natural chemical sorption and transformations when P fertilizer is applied to soils. A perplexing challenge facing agriculture production is finding sustainable solutions to deliver P more efficiently to plants. Using prescribed applications of specific soil microbial assemblages to mobilize soil bound—P to improve crop nutrient uptake and productivity has rarely been employed. We investigated whether inoculation of soils with a bacterial consortium developed to mobilize soil P, named Mammoth P^TM^, could increase plant productivity. In turf, herbs, and fruits, the combination of conventional inorganic fertilizer combined with Mammoth P^TM^ increased productivity up to twofold compared to the fertilizer treatments without the Mammoth P^TM^ inoculant. Jalapeño plants were found to bloom more rapidly when treated with either Mammoth P. In wheat trials, we found that Mammoth P^TM^ by itself was able to deliver yields equivalent to those achieved with conventional inorganic fertilizer applications and improved productivity more than another biostimulant product. Results from this study indicate the substantial potential of Mammoth P^TM^ to enhance plant growth and crop productivity.

## Introduction

In the 1960s the “Green Revolution” averted a potentially catastrophic lack of food production in the developing world (Khush, 2001; Lam, 2011) allowing the global population to double while still meeting food demands (Lam, 2011; Tilman, 1998). Most of the agricultural gains over the last fifty years have been accomplished through a combination of management approaches such as irrigation, plant genetic manipulation and breeding efforts, and a surge in nitrogen and phosphorus fertilizer usage (Conant, Berdanier & Grace, 2013; Lam, 2011). However, current agriculture production is insufficient to provide enough food for the growing global population through the mid-21st century (Abelson, 1999; Ray et al., 2013).

Phosphorus (P) is a finite resource with substantial resources found in only 10 countries (Cordell, 2010; Jasinski, 2013); and peak global phosphorus availability could occur in less than three decades (Craswell et al., 2010; Steen, 1998). Phosphorus is a critical nutrient used to maximize plant growth and yield. Thus, reductions in the supply of P fertilizers could severely diminish crop yields. One solution for mitigating the threat of this diminishing resource is to develop sustainable technologies to improve P use efficiently for plant uptake.

An equally perplexing challenge facing agriculture production is finding solutions to efficiently deliver P to plants (MacDonald et al., 2011). Current agriculture management practices commonly experience low plant nutrient use efficiency due to natural chemical sorption and transformations when fertilizer is applied to soils. For example, up to 90% of P fertilizer applied to soil is made unavailable to plants because it binds to Ca, Al and Fe- bearing soil mineral surfaces or is lost from the ecosystem by leaching (Doolette & Smernik, 2011; Randriamanantsoa et al., 2013). Soils with a large capacity to bind P are especially concerning because P delivery to plants requires more inputs relative to the P outputs in harvested crops (MacDonald et al., 2011), resulting in extremely low agricultural phosphorus use efficiency.

Soil bacteria can strongly influence the amount of soil P that is plant-available by solubilizing the mineral-associated P (Malboobi et al., 2009; Osorio & Habte, 2014; Tawaraya, Naito & Wagatsuma, 2006) and increasing plant P uptake by stimulating plant root growth (Bal et al., 2013; Penrose & Glick, 2003; Rashid, Charles & Glick, 2012). They achieve this by releasing metabolites including organic acids and high-affinity iron chelating siderophores which solubilize mineral-bound P (Richardson et al., 2009; Shropshire & Bordenstein, 2016) and by exuding plant hormones such as auxin (Spaepen, 2015). Since there are multiple mechanisms by which microbes can solubilize P, microbial consortia (multiple species) may be more effective than single species isolates because no single strain is likely to be optimized for the many various mechanisms that drive this process. Previous studies have shown that a consortium of P-mobilizing bacteria are more effective at making P available than the individual member species (P Baas et al., 2016, unpublished data) and a few other studies have provided evidence for synergistic effects between multiple microbial species (Kim, Jordan & McDonald, 1997; Tarafdar & Marschner, 1995). Might a consortium of P-mobilizing bacteria improve processes like plant emergence, blooming, and productivity?

The use of microbial biostimulants offers the promise of improved microorganism activities to enhance plant growth (Richardson & Simpson, 2011). One of these products, Mammoth P™ (Growcentia, Fort Collins, CO, USA), is a comprised of a novel microbial consortium selected for its superior capacity to solubilize soil P. In this study, we tested the effect of Mammoth P™ to increase plant emergence, blooming, and productivity for a variety of plant species. Our objective was to test the efficacy of this microbial biostimulant to enhance plant growth across a wide variety of crops, including: wheat, herbs, fruits and turf grass. We predicted that the addition of a consortia of microbes found in Mammoth P™ would increase plant productivity, whereas the greatest effect would occur when the plant is both fertilized and inoculated with Mammoth P™. We further predicted that secondary metabolites produced by the microbes—but in the absence of active microbes—could have a positive effect on plant performance.

## Materials and Methods

### The inoculum

We tested the effect of the Mammoth P™ on plant growth, flowering, and fruit productivity. Mammoth P™ is comprised of a consortium of four bacterial taxa (*Enterobacter cloacae*, *Citrobacter freundii*, *Pseudomonas putida* and *Comamonas testosteroni*; Table 1) by a proprietary method that selected for the ability to mobilize bound P. Mammoth P™ is a liquid culture that we applied directly to the soil. Bacterial cultures were grown in a proprietary, P-limiting media (C:N:P:K = 1:2:108:29; Growcentia Inc., Fort Collins, CO, USA) for three days reaching at least 10^8^ colony forming units (CFU) ml^−1^ (Black, 2008). The specific bacterial species were determined using the streak plating technique, amplified with a 16S 15f/1492f primer set and Sanger sequenced at the Proteomics and Metabolomics Facility at Colorado State University (Black, 2008).

**Table 1:** The relative proportions (%) of the top four species representing >95% of all operationally defined units (OTU).

Family	Genus/species	Abundance (%)
*Enterobacteriaceae*	*Citrobacter freundii*	35 ± 4
*Enterobacteriaceae*	*Enterobacter cloacae*	17 ± 2
*Pseudomonadaceae*	*Pseudomonas putida*	38 ± 6
*Comamonaceae*	*Comamonas testosteroni*	6 ± 2
Total		96 ± 1

### Experimental design

Plant productivity was assessed in multiple greenhouse trials for hard red winter wheat (*Triticum aestivum*), fescue turf grass (*Festuca arundinacea*; Kentucky 31 variety), jalapeño (*Capsicum annuum*; early jalapeño variety), cherry tomato (*Solanum lycopersicum*; Sweetie variety) and basil (*Ocimum basilicum*; Italian Genovese variety). We tested plant performance with fertilizer, Mammoth P™, metabolites produced by the Mammoth P™ consortium, and interactions among the treatments. Treatments varied and included some or all of the following seven: (1) P-limiting media (media control); (2) inoculation with Mammoth P™; (3) Mammoth P™ metabolites; (4) fertilizer only; (5) Mammoth P™ plus fertilizer; (6) Mammoth P™ metabolites plus fertilizer and (7) water. Mammoth P™ metabolites were collected by filtering culture through a 0.2-micron filter. Fertilization rates were conducted following manufacturer’s recommendations. Plants in the greenhouse were watered daily and the temperature was 22 ± 2.5°C with supplemental growth lights running a total of 16 h a day. We used a Completely Randomized Design (CRD) to assign specific plants to treatments.

We used two varieties of red hard winter wheat (Byrd and Hatcher) and two types of soil from (1) the Agricultural Research Development & Education Center (ARDEC) and (2) Waverly, a 130 ha Colorado State University managed property located north of Fort Collins, Colorado (40°42′54″N, 105°50′53″W). The wheat was vernalized for 6 weeks at 7°C and the seedlings were subsequently planted into planter trays. After two weeks the plants were transplanted to a 3.8 L pot containing the same Waverly and sand mixture or ARDEC and sand mixture. The pots were filled with 2 mm sieved Waverly soil or 4 mm sieved ARDEC soil mixed (1:1) with washed sand. Plants were treated with either Mammoth P™, Mammoth P™ metabolites, Hoagland’s solution or water at the initial planting of the seedlings (1 mL pot^−1^), at transplant (5 mL pot^−1^) and a third time 2 weeks after transplanting (5 mL pot^−1^). To assess the efficacy of Mammoth P™ compared to another biostimulant currently on the market we also included a treatment of Accomplish LM (Agricen Inc., Loveland, CO, USA). After two months the plants were harvested for aboveground biomass. Plant material was dried at 65°C until at constant weight to determine total plant dry biomass.

Fescue turf grass was planted in a Waverly-sand mixture in planting trays (0.7 L & 1.4 L). We tested the seven treatments described above excluding the P-limiting media treatment. Mammoth P™ inoculation was done at planting with 30 mL per .56 g of seed. The fertilizer treatment was done at planting using the slow-release fertilizer formulation Scotts Turf Starter following the manufacturer’s recommendations (The Scotts Company LLC, Marysville, OH, USA). After two months the aboveground biomass was collected, dried at 65°C and weighted for aboveground productivity.

The herb and fruit experiments were conducted using 4 mm sieved soil from the Agricultural Research Development & Education Center (ARDEC) mixed 1:1 with Fafard 4P potting soil (Sun Gro Horticulture Inc., Agawam, MA, USA). First we planted cherry tomato, basil, and jalapeño seeds in 30 ml of soil mixture using a planter tray. After 7 weeks from planting all seedlings were transplanted to a 1 L pot and after 3 months from planting the cherry tomatoes were transplanted to a 7.6 L pot. To provide a baseline level of nutrient availability, all plants were fertilized weekly for the first month after transplanting using half the recommended level of fertilization with Jack’s classic 20:20:20 fertilizer (125 mg L^−1^) and weekly (i.e., tomato) or twice monthly applications (i.e., basil and jalapeño) with 100 mg L^−1^ N 15-5-15 Technigro (Sun Gro Horticulture Inc., Agawam, MA, USA). These fertilization regimes represent a minimal basal level of fertilization (Heeb et al., 2005). The jalapeño and basil plants designated to receive inoculum were inoculated at planting, after 1, 1.5, 2, and 3 months in addition to immediately after transplanting. The cherry tomato plants were inoculated with Mammoth P™ after planting, transplanting and 1, 1.5, and 3 months. The treatment volume was 2 mL pot^−1^ (planter) and 5 mL pot^−1^ (1 L and 7.6 L pot). The fertilized treatments received the slow release formulation Miracle Gro Shake ‘n Feed^®^ (The Scotts Company LLC, Marysville, OH, USA), as recommended, at planting and after 5 months. Basil plants were cut back to the top four starter leaves to prevent flowering and, thus, maximize leaf productivity. Fresh weight of basil leaves collected was used to determine yield. Jalapeno buds and blooms were counted twice monthly and jalapeno peppers were harvested when 5 cm was reached or the pepper had turned red. Fresh weight of peppers was used to determine yield. After 6 months we harvested the remaining peppers and basil leaves and determined a terminal yield by fresh weight of harvest. For cherry tomato plants, we determined the number of buds, blooms and red tomatoes twice monthly for 4 months after planting.

### Statistics and calculations

We determined treatment differences using analysis of variance analyses (ANOVA) with multiple comparisons using Tukey tests. The vegetable data were analyzed using a repeated measures approach with orthogonal contrasts to determine treatment differences. Data was tested for normality using Q–Q plots and if proven non-normal, were log transformed. Significant differences indicate *p* < 0.05 unless stated otherwise. All statistics were conducted in SAS JMP 11.0.

## Results

### Plant emergence and bloom development

**Figure 1:**
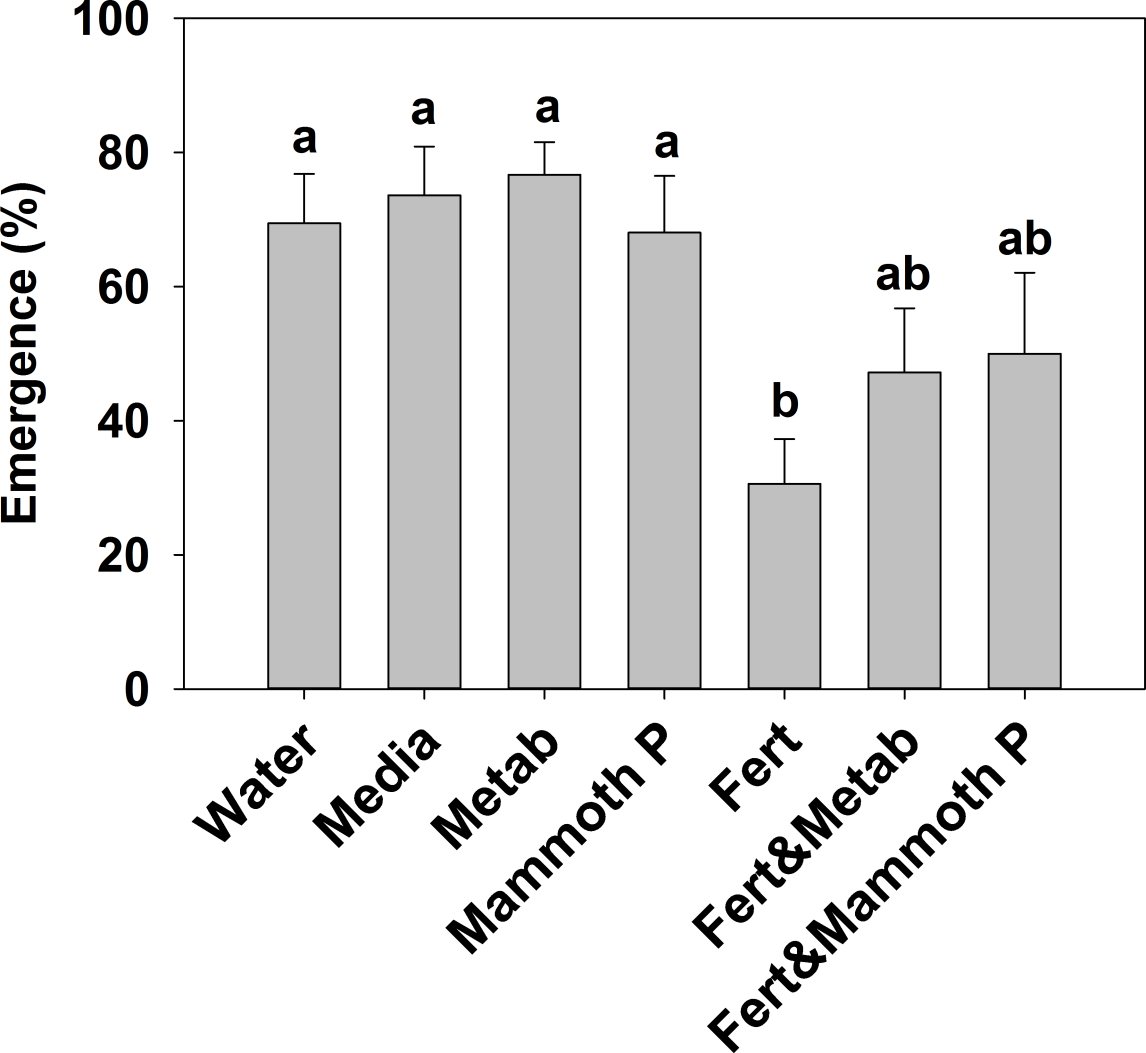
Plant emergence for basil, broccoli, jalapeño, marigold flowers and tomatoes (brandy wine and cherry varieties). Different letters indicate significant differences. Water, the water control; Metab, culture metabolites; Fert, fertilized with Hoagland’s solution; Mammoth P, inoculated with Mammoth P.

The addition of a slow release fertilizer significantly reduced plant emergence by 55–50% compared to the Mammoth P™, metabolite, media, and water treatment (Fig. 1). The proportional increases in emergence of the fertilizer plus Mammoth P™ (+89 ± 44%) and the fertilizer plus metabolite (+108 ± 70%) treatments compared to the fertilizer-only treatment were significantly greater than zero (*p* < 0.001).

**Figure 2:**
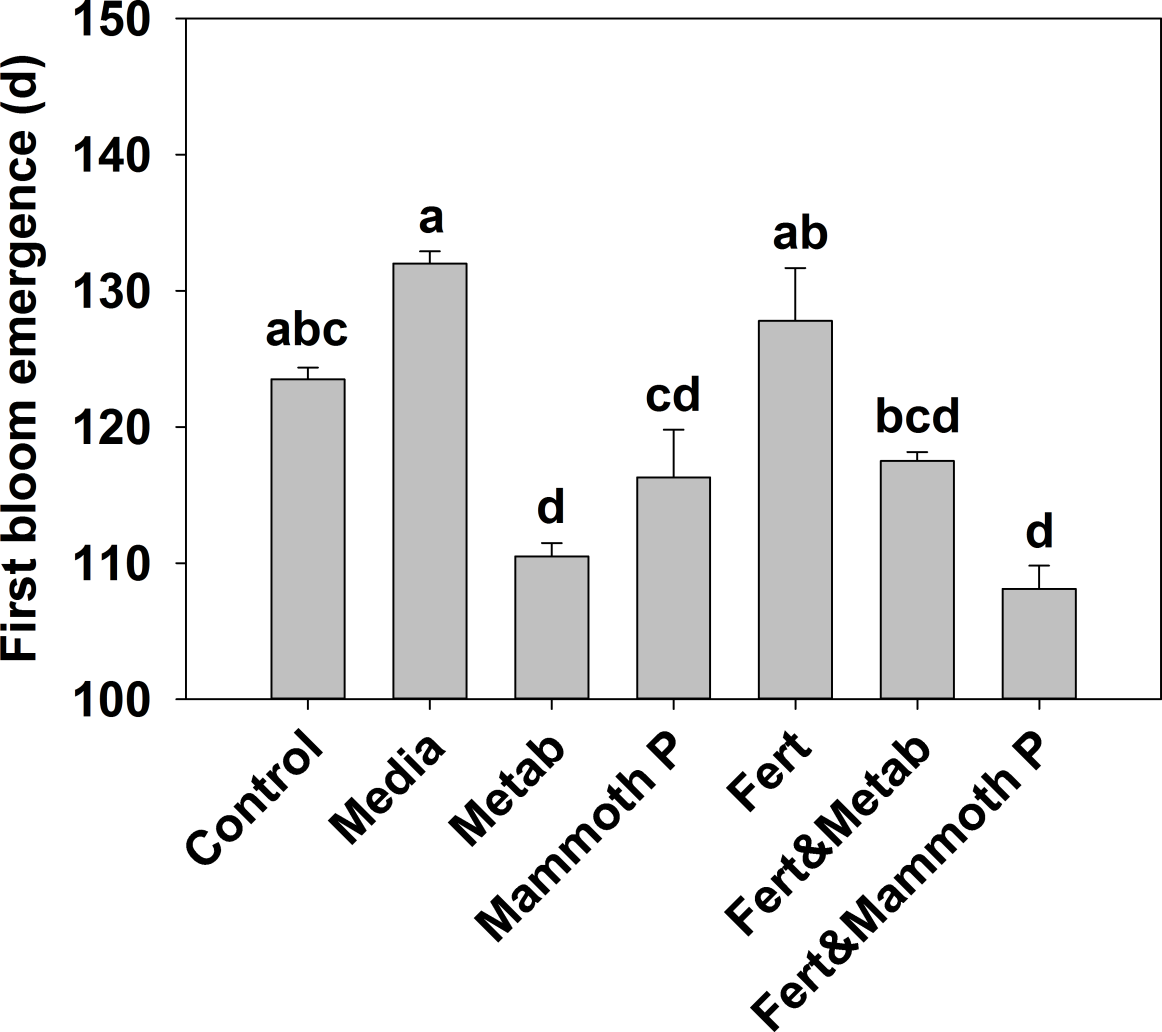
Time for the development of the first bloom in jalapeño plants. Different letters indicate significant differences. Control, water control; Metab, culture metabolites; Fert, fertilized with Scotts Turf Starter; Mammoth P, inoculated with Mammoth P.

Jalapeño plants showed that the time to develop blooms was significantly lower for the Mammoth P™ (9%), metabolite (14%), and Mammoth P™ plus fertilizer (16%) treatments compared to the fertilizer only treatment (Fig. 2). Additionally, the fertilized treatment showed no accelerated bloom development compared to the control or media control treatments. Combining Mammoth P™ with a fertilizer significantly reduced the time to first bloom while the metabolite treatment with fertilizer did not show a reduced time to first bloom.

### Plant productivity

Plant productivity was generally significantly greater if Mammoth P™ was applied compared to the fertilizer only treatments. The greatest improvements in productivity compared to the fertilized treatment were found for the Mammoth P™ plus fertilizer treatments, with increased productivity of up to 91%. Additionally, Mammoth P™ metabolites plus fertilizer treatments often had positive effects on productivity similar to the Mammoth P plus fertilizer treatment.

**Figure 3:**
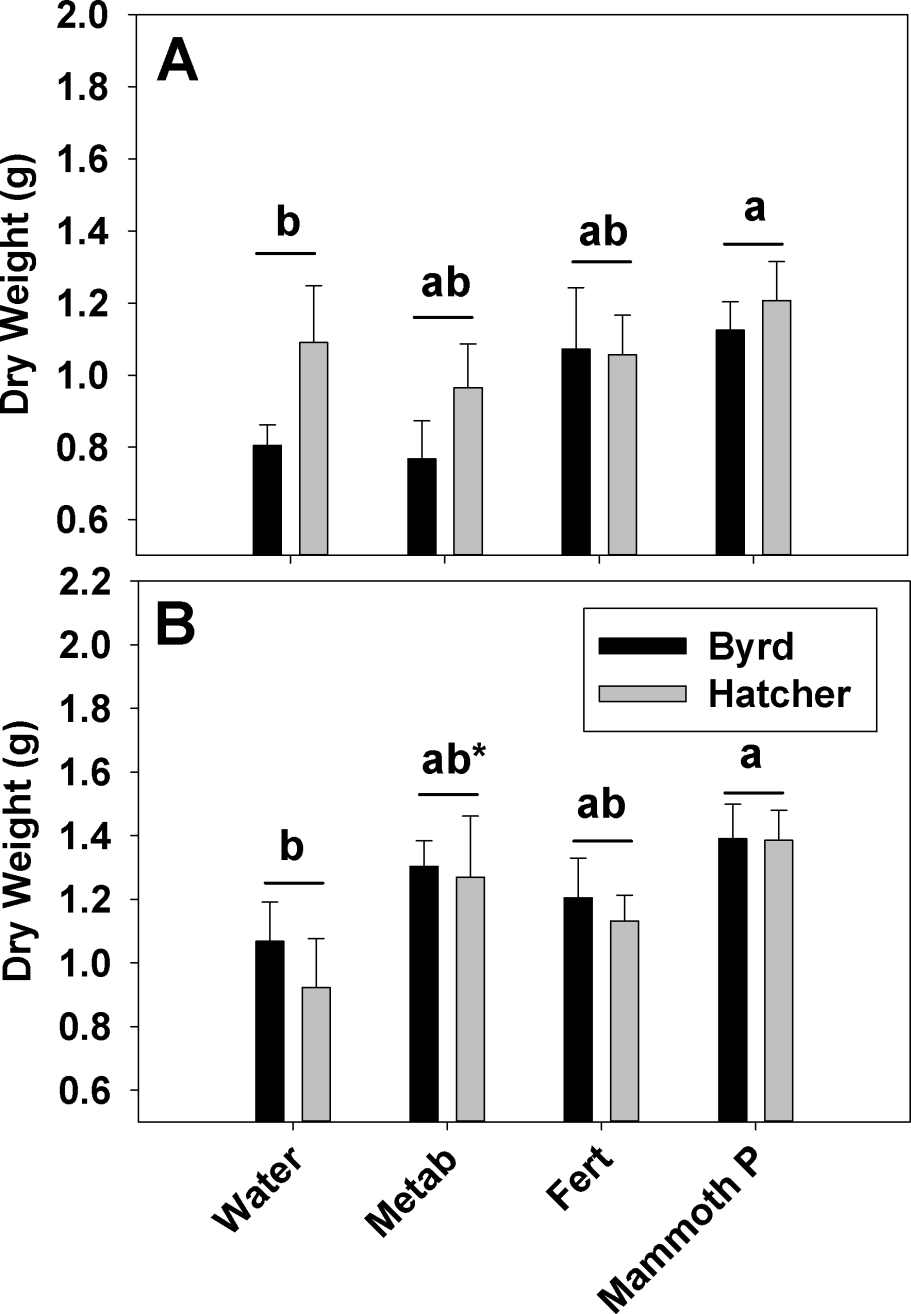
Red hard winter wheat aboveground biomass after a two months growth period. Aboveground biomass data is shown for the Waverly (A) and ARDEC (B) soil types. Different letters indicate significant differences. Water, the water control; Metab, culture metabolites; Fert, fertilized with Hoagland’s solution; Mammoth P, inoculated with Mammoth P. *different from the water control at *p* = 0.06.

**Figure 4:**
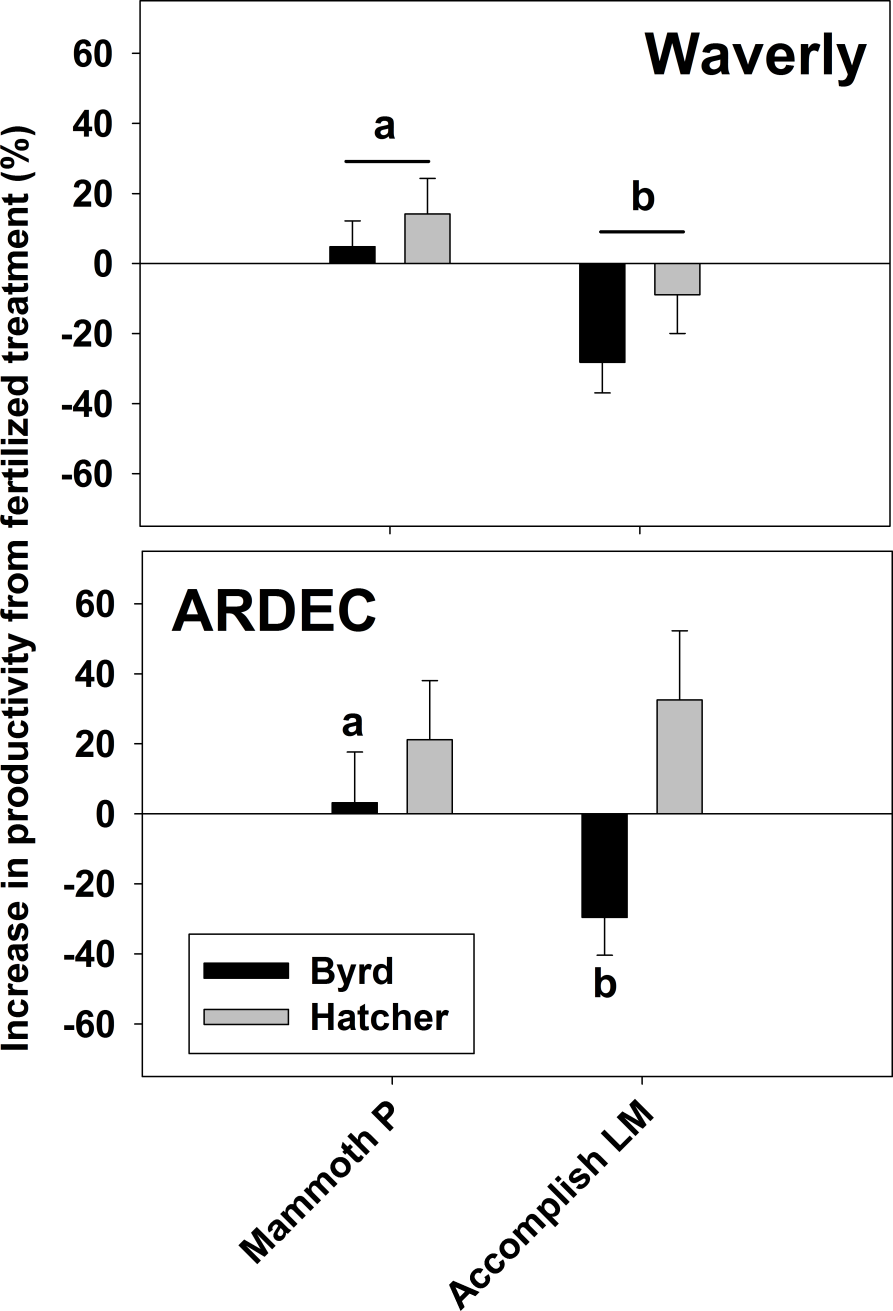
Fescue turf grass aboveground biomass two months after seeding. The bars indicate the mean and the error bars indicate the standard error with different letters indicate significant differences. Water, the water control; Metab, culture metabolites; Fert, fertilized with Hoagland’s solution; Mammoth P, inoculated with Mammoth P. *different from the water control at *p* = 0.09.

Hard red winter wheat productivity (Fig. 3) for the fertilized, metabolites, and Mammoth P™ treatments ranged from 0.77 to 1.4 g plant^−1^ and the increase in productivity from the mean water control was greatest in the Mammoth P™ treatment (41 ± 6%) followed by the fertilized (23 ± 9%) and the metabolite treatment (16 ± 7%). Overall, the Mammoth P™ treatment was significantly greater than the water control and in the ARDEC soil type the metabolite treatment exhibited greater productivity. Compared to another biostimulant product, Accomplish LM™, Mammoth P™ improved productivity significantly more in Waverly soil and, for the Byrd variety, in ARDEC soil (Fig. 4). No differences in productivity improvement were found between the two products for the Hatcher variety in ARDEC soil.

**Figure 5:**
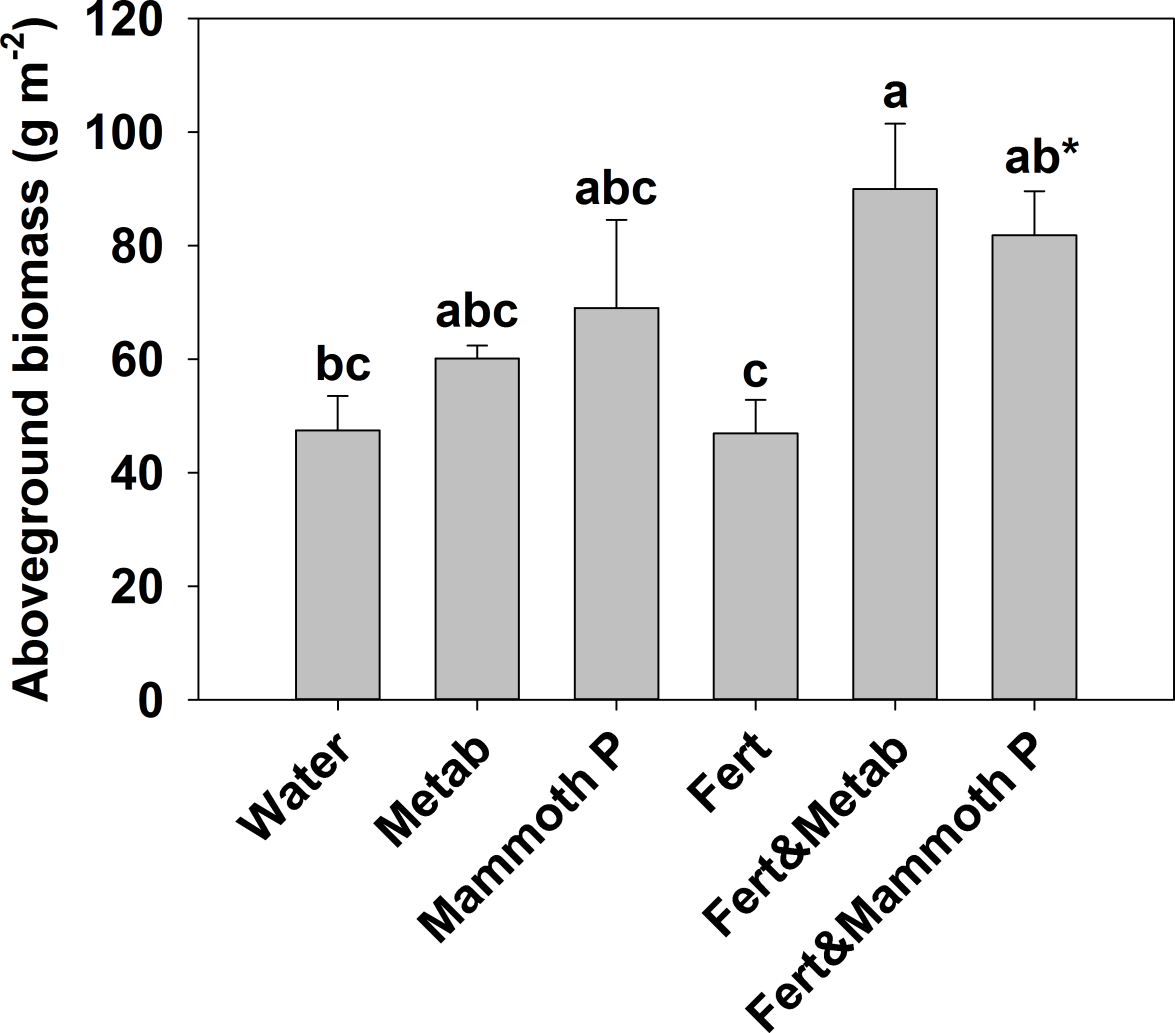
Plant productivity over time for the cumulative basil leaf (a), cumulative jalapeño peppers (b) and total number of cherry tomato fruits (c). The points indicate the mean and the error bars indicate the standard error. Control, water control; Media, sterile media; Metab, culture metabolites; Fert, fertilized; MP, inoculated with Mammoth P.

Fescue turf grass productivity (Fig. 5) was greatest when Mammoth P™ or its metabolites were combined with fertilization with productivity being 74–91% greater than traditional fertilization practices. The fertilizer plus Mammoth P™ and fertilizer plus metabolite treatments were significantly greater than both the water control (*p* = 0.9 for metabolite & fertilizer) and the fertilized treatment.

**Figure 6:**
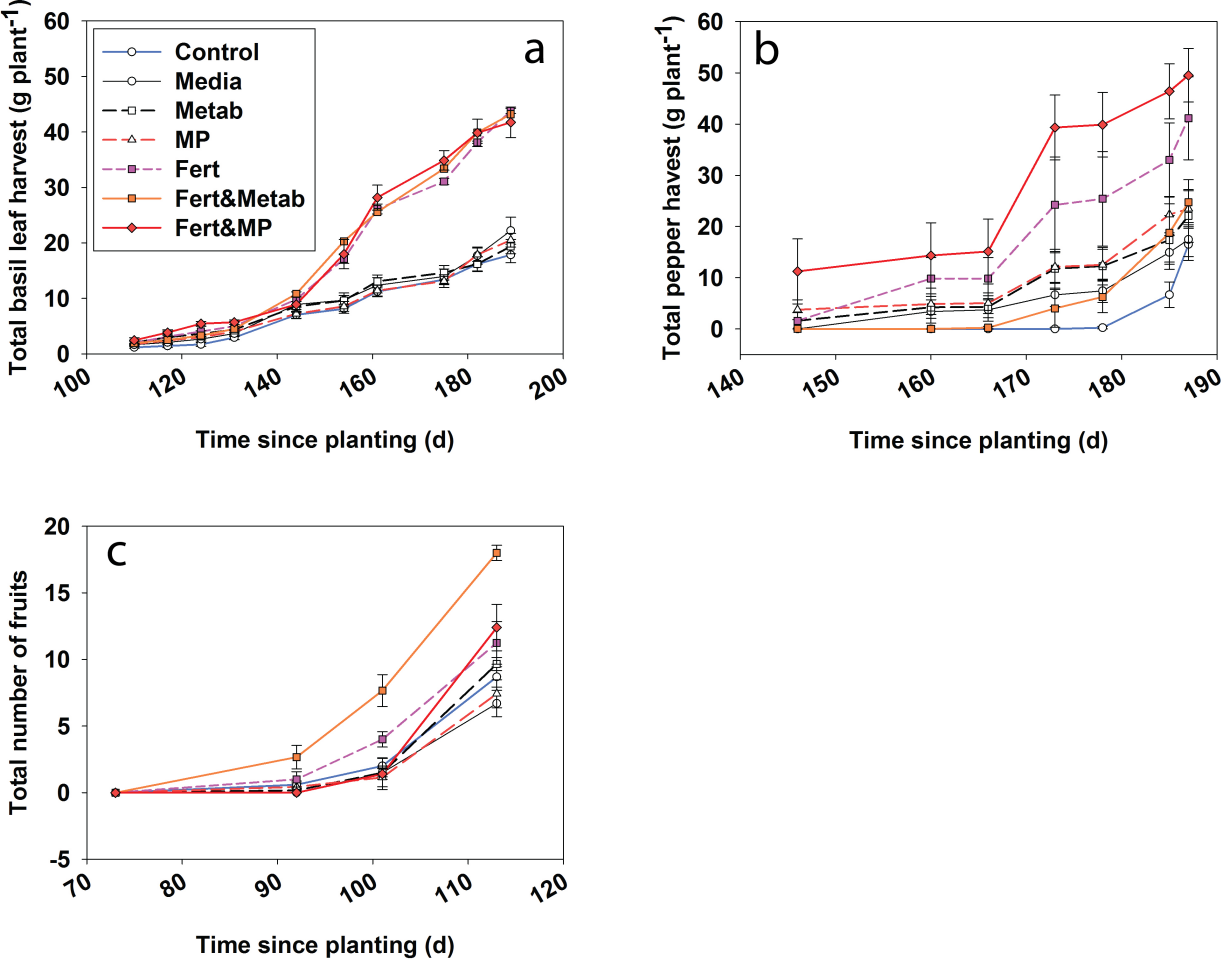
The increase in productivity from the fertilized treatment for red hard winter wheat. Data is shown for the Waverly (A and B) and ARDEC (C) soil types. Different letters indicate significant differences (*p* < 0.1). Mammoth P, inoculated with Mammoth P; accomplish LM, amended with Accomplish LM by Agricen, Inc.

Herb and fruit productivity (i.e., basil, jalapeño and tomato) showed significant treatment effects over time. After 6 months of growth the jalapeño productivity (Fig. 6) for the Mammoth P™ plus fertilizer treatment were significantly different (41 ± 16%) compared to the fertilizer treatment. Mammoth P™ inoculation plus fertilizer was not significantly different from the fertilizer treatment for jalapeño and basil while marginally significant for tomato (*p* < 0.1). Basil productivity in the fertilized treatments with or without Mammoth P™ or metabolites were significantly greater than the remaining treatments. Jalapeño productivity was significantly greater with Mammoth P™ plus fertilizer than the water control treatments. Tomato productivity was greater for the Mammoth P™ plus fertilizer (*p* < 0.09) and the metabolite plus fertilizer (*p* < 0.001) treatments.

## Discussion

The use of the microbial biostimulant Mammoth P™ dramatically improved plant emergence and productivity across a wide variety of plant species and soil types over what can be achieved with fertilizer alone. These results suggest that using sustainable microbial biostimulant technologies such as this can stimulate bloom and improve plant production, potentially providing a sustainable solution to meet the food, fiber and fuel needs of a growing population.

We found that Mammoth P™ inoculations resulted in higher plant productivity compared to traditional fertilizer treatments. We also found that Mammoth P™ enhanced productivity when combined with fertilizer treatments. In part, this is likely due to the natural nutrient cycling processes that facilitate plant nutrient uptake. Previous studies have shown that up to 50% of ecosystem productivity is due to plant interactions with a variety of bacterial, archaeal, and mycorrhizal species (Adesemoye & Kloepper, 2009; Hassan et al., 2013; Van Der Heijden, Bardgett & Van Straalen, 2008). Our findings also confirm the conceptual framework proposed by Van Der Heijden, Bardgett & Van Straalen (2008) suggesting that microbial inoculation may be most effective under lower nutrient availability, as indicated by the turf and wheat trails.

The lower emergence with fertilizer-only treatments was surprising but has been observed before (Raun, Sander & Olson, 1986; Rehm & Lamb, 2009). The magnitude and direction of the fertilizer effect is likely determined by the soil type (Rehm & Lamb, 2009). Shortening time-to-bloom by 16% in jalapeño plants for both the metabolite and Mammoth P™ treatments suggest that microbial products may produce a more favorable environment for plant development. We speculate that the usage of a multi-species inoculum allows the consortium to function under a wide variety of environmental conditions while maintaining efficacy. We hypothesize that the interactions among the inoculum’s microbial constituent species resulted in the formation of metabolites capable of enhancing the plant investment in fruit development.

Mammoth P™ additions successfully increased productivity among a wide variety of crop species and also showed more consistent results for wheat productivity than another commercially available biostimulant, Accomplish LM™. Efficacy reports of Accomplish LM™ reported flowering increases of 26–40% (sunflower) and 2–14% (*chrysanthemum*) with productivity increases between 5 and 20% (Savov, Chakalov & Popova, 2011; Savov, Chakalov & Popova, 2012). We found that the inoculation of the four-species consortia in Mammoth P™ enhanced productivity by up to 91%. Previous studies have found positive effects of inoculation on the rate of emergence (Kropp et al., 1996; Lucy, Reed & Glick, 2004) which been linked to increased root growth (Chanway, Radley & Holl, 1991). But to our knowledge, this phenomenon has never been observed in such a wide variety of plants.

Microbial metabolites could increase plant allocation to bloom development by being rich in compounds capable of solubilizing P and micronutrients by reducing the soil pH (Khan et al., 2009), by producing extracellular enzymes capable of liberating nutrients (Khan et al., 2009), or by acting as a plant hormone. Indeed, microbial produced metabolites such as salicylic acid, ethylene, glutamate, auxins and many more have been linked to increased plant disease resistance (Van Wees, Van der Ent & Pieterse, 2008), growth promotion (Marks et al., 2015; Spaepen, 2015), stimulating the induction of flowering (Raskin, 1992) and increasing vitamin uptake (Yousaf et al., 2015). Basil showed no effect from Mammoth P™ or its metabolites and it is likely the plants were already functioning at maximum capacity with generally lower nutrient requirements than fruits (Sharafzadeh & Alizadeh, 2011; Tesi et al., 1994).

Microbial nutrient immobilization is important in preventing nutrient loss when plant uptake is slow or inactive, whereby the microbial biomass can function as a “slow-release fertilizer” at later plant developmental stages (Malik, Khan & Marschner, 2013; Singh et al., 1989). Our results suggest the importance of microbial products on influencing plant productivity. Plant exudation likely influences microbial communities (Brimecombe, De Leij & Lynch, 2000) and may determine whether plants and the proximal soil microbes engage in symbiotic, neutral or competitive relationships (Spaepen, 2015). Some plant species may support a beneficial relationship with the inoculum bacterial community (i.e., jalapeño, wheat and turf) while others mainly respond to microbial metabolites such that live microbes might reduce the metabolite effect by competing for resources with the microbial community (i.e., tomato). Overall, our results suggest that microbial communities might be more in control of microbial-plant interactions than previously recognized.

## Conclusions

Overall, Mammoth P™ inoculations facilitated superior plant emergence, faster blooming and plant productivity across a wide range of crop species. Our results suggest that microbial metabolites may play a crucial role in controlling plant growth and that the microbial community might be controlling plant development in a variety of ways. These results indicate the vast potential for future development of consortia-based inocula to transform agriculture.

##  Supplemental Information

10.7717/peerj.2121/supp-1 Table S1 Raw data table Detailing the data collected from the different greenhouse experiments.

## Additional Information and Declarations

### Data Availability

The following information was supplied regarding data availability: The raw data has been supplied as Table S1.
